# Anti-Inflammatory Activity of Bee Venom in BV2 Microglial Cells: Mediation of MyD88-Dependent NF-*κ*B Signaling Pathway

**DOI:** 10.1155/2016/3704764

**Published:** 2016-08-03

**Authors:** Eun Ju Im, Su Jung Kim, Seung Bok Hong, Jin-Kyu Park, Man Hee Rhee

**Affiliations:** ^1^Laboratory of Veterinary Physiology and Cell Signaling, College of Veterinary Medicine, Kyungpook National University, Daegu 41566, Republic of Korea; ^2^Department of Biomedical Laboratory Science, Daegu Health College, Daegu 41453, Republic of Korea; ^3^Department of Clinical Laboratory Science, Chungbuk Health and Science University, Chenogju 28150, Republic of Korea; ^4^Beesen R & D Institute, Beesen Co., Ltd., Daejeon 34054, Republic of Korea

## Abstract

Bee venom has long been used as a traditional folk medicine in Korea. It has been reportedly used for the treatment of arthritis, cancer, and inflammation. Although its anti-inflammatory activity in lipopolysaccharide- (LPS-) stimulated inflammatory cells has been reported, the exact mechanism of its anti-inflammatory action has not been fully elucidated. Therefore, the aim of this study was to investigate the anti-inflammatory mechanism of bee venom in BV2 microglial cells. We first investigated whether NO production in LPS-activated BV2 cells was inhibited by bee venom, and further iNOS mRNA and protein expressions were determined. The mRNA and protein levels of proinflammatory cytokines were examined using semiquantitative RT-PCR and immunoblotting, respectively. Moreover, modulation of the transcription factor NF-*κ*B by bee venom was also investigated using a luciferase assay. LPS-induced NO production in BV2 microglial cells was significantly inhibited in a concentration-dependent manner upon pretreatment with bee venom. Bee venom markedly reduced the mRNA expression of COX-2, TNF-*α*, IL-1*β*, and IL-6 and suppressed LPS-induced activation of MyD88 and IRAK1 and phosphorylation of TAK1. Moreover, NF-*κ*B translocation by IKK*α*/*β* phosphorylation and subsequent I*κ*B-*α* degradation were also attenuated. Thus, collectively, these results indicate that bee venom exerts its anti-inflammatory activity via the IRAK1/TAK1/NF-*κ*B signaling pathway.

## 1. Introduction

Toll-like receptors (TLRs) are highly expressed in microglial cells, and they are involved in the functioning of the innate inflammatory response to a wide range of invading microorganisms by releasing proinflammatory cytokines and chemokines [1]. One of these TLRs, that is, TLR4 which can be rapidly activated by lipopolysaccharide (LPS), acts as a potent activator and initiates the inflammatory cascade in cells [2]. Consequently, activation of TLR4 triggers its association with downstream adopter molecules within the cytoplasm, such as myeloid differentiation primary response gene 88 (MyD88), and subsequent association with interleukin-1 receptor-associated kinase 1 (IRAK1). This leads to activation of the transcription factor, that is, nuclear factor-kappa B (NF-*κ*B) [3].

In the central nervous system (CNS), microglia, which are macrophage-like innate immune cells, play a crucial role in host defense mechanisms and tissue repair [4]. Under pathological conditions such as brain tissue injury or in the presence of immunological stimuli, microglia are rapidly activated and produce inflammatory mediators such as tumor necrosis factor-*α* (TNF-*α*), interleukin 6 (IL-6), and IL-1*β* [5]. Expression of these inflammation-related cytokines is regulated at the transcriptional level. NF-*κ*B is a key transcriptional regulator of inflammatory cytokine expression in the immune cells. Accumulation of inflammatory cytokines can cause severe neurodegenerative diseases, including Alzheimer's disease, Parkinson's disease, and trauma [6–8]. Therefore, suppression of these inflammation-related mediators is particularly important for the prevention of neurodegenerative diseases in the CNS.

Bee venom, which is extracted from honeybees, has long been used in alternative medicine and as a traditional Korean folk medicine [9]. A number of studies have shown that bee venom has diverse physiological activities such as antiarthritic [10], anticancer [11], and anti-inflammatory action [9]. Bee venom contains melittin, apamin, adolpin, and mast cell-degranulating peptide [12]. Recent reports have shown that two of the main constituents of bee venom, melittin and apamin, have anti-inflammatory effects on LPS-stimulated BV2 microglial cells through p38 mitogen-activated protein kinase (MAPK) and NF-*κ*B-dependent signal transduction [9]. Although several studies on the bioactivities of bee venom have been conducted, the actual molecular mechanism by which bee venom regulates inflammation and the signaling pathway has not yet been completely elucidated.

In the present study, in order to determine the mechanism underlying the anti-inflammatory effects of bee venom in BV2 microglia, we investigated its inhibitory effect on the expression of proinflammatory cytokines and the associated molecular signaling pathways. We established that bee venom could attenuate the expression of proinflammatory mediators through a MyD88-dependent signaling pathway.

## 2. Materials and Methods

### 2.1. Materials

Dulbecco's Modified Eagle's Medium (DMEM) and fetal bovine serum (FBS) were obtained from Welgene (Daegu, South Korea). Streptomycin and penicillin were obtained from Lonza (MD, USA). TRI Reagent® solution (AM9738) was obtained from Applied Biosystems/Ambion (Warrington, UK); Oligo(dT) primers were obtained from Bioneer oligo synthesis (Daejeon, Korea). SYBER® green master mix was obtained from Applied Biosystems (Warrington, UK). iNOS, COX-2, TNF-*α*, and IL-1*β* primers were obtained from Bioneer (Daejeon, Korea). Total protein lysis buffer (PRO-PREP) and the PRO-MEASURE protein assay kit were obtained from iNtRON Biotechnology (Seoul, Korea). LPS (*Escherichia coli* 055:B5) and 3-(4,5-dimethylthiazol-2-yl)-2,5-diphenyltetrazolium bromide (MTT) were purchased from Sigma (St. Louis, MO, USA). Specific antibodies against phosphor and/or total forms of ERK, JNK, p38, IKK*α*/*β*, I*κ*B, NF-*κ*B p65, PI3K, Akt, PARP, iNOS, COX-2, HO-1, and *β*-actin, as well as a rabbit HRP-conjugated antibody, were purchased from Cell Signaling Technology (Danvers, MA, USA). All other reagents and chemicals were obtained from Sigma Aldrich (St. Louis, MO, USA).

### 2.2. Bee Venom Preparation

**Figure 1:**
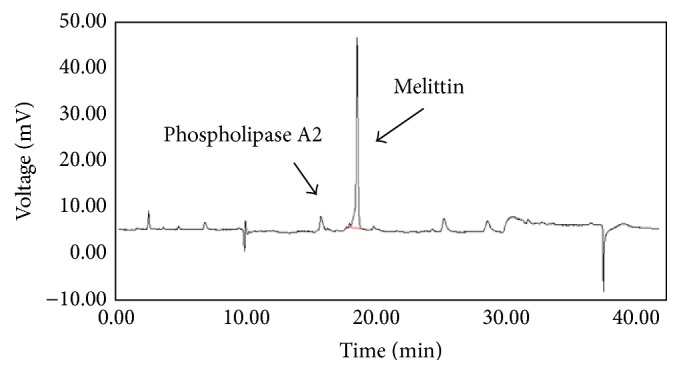
HPLC analysis of bee venom extract used in this experiment.

The crude honeybee venom was obtained using Large Quantity Bee Venom Collector (P10-1003672, Wissen Co., Ltd., Daejeon, Korea). The bee venom (2.5 g) was solubilized with 250 mL ultrafiltered water and filtered through a 0.45 *μ*m nylon membrane filter (Millipore, Billerica, MA, USA) under vacuum. The filtrates were dried using a freeze dryer (Han IL Sci., Clean Vac 8). The dried powder (10 mg) was dissolved in 1 mL of ultrafiltered water and analyzed by HPLC (Figure 1). The contents of melittin and phospholipase-2 were found to be 59.62 ± 4.25% and 12.03 ± 0.66%, respectively.

### 2.3. Cell Culture

BV2 microglia were maintained in DMEM enriched with 10% heat-inactivated FBS, 100 *μ*g/mL streptomycin, and 100 U/mL penicillin and incubated in a humidified atmosphere of 5% CO_2_ at 37°C [13].

### 2.4. Nitric Oxide Determination

Nitric oxide (NO) in the culture supernatant was measured as previously described [14]. Briefly, BV2 microglia cultured in 24-well plates were incubated with or without LPS (0.1 *μ*g/mL) in the absence or presence of bee venom at the indicated concentrations for 18 h. The cell culture supernatants (100 *μ*L) were mixed with Griess reagent (1% sulfanilamide in 5% phosphoric acid [H_3_PO_4_] and 0.1% N-(1-naphthyl)ethylenediamine dihydrochloride [NEDHC]) and incubated for 5 min at room temperature. The absorbance was measured at 540 nm in a microplate reader.

### 2.5. Cell Viability Assay

BV2 microglial cells were seeded in 24-well plates and treated with or without LPS (0.1 *μ*g/mL) in the absence or presence of various concentrations of bee venom in culture medium. After 18 h, the BV2 microglial cells were incubated with 3-(4,5-dimethylthiazol-2-yl)-2,5-diphenyltetrazolium bromide (MTT) reagent for 4 h, following which the absorbance was measured at 560 nm in an ELISA reader.

### 2.6. RNA Extraction and Quantitative PCR

BV2 microglial cells were pretreated with various concentrations of bee venom for 30 min and then stimulated with LPS (0.1 *μ*g/mL) for 18 h. Total RNA was isolated using TRI Reagent solution according to the manufacturer's instructions. Total RNA (2 *μ*g) was reverse transcribed using reverse transcriptase premix and oligo(dT) primers. Quantitative PCR was performed with the CFX96*™* Real-Time System (Bio-Rad) by using power SYBR® Green Master Mix. The relative quantity of target mRNA was calculated using the comparative threshold (Ct) method by normalizing to GAPDH Ct values. The quantitative PCR program used was as follows: predenaturation (95°C, 5 min), denaturation (95°C, 20 sec), annealing (55°C, 20 sec), and extension (72°C, 45 sec), using primers specific for* iNOS*,* COX-2*,* IL-6*, and* TNF-α*.

### 2.7. Western Blotting

BV2 microglial cells were pretreated with various concentrations of bee venom for 30 min in DMEM. After incubation with LPS (0.1 *μ*g/mL), BV2 microglial cells were washed and scraped in ice-cold phosphate-buffered saline (PBS), and the cell pellets were resuspended in lysis buffer (PRO-PREP) containing 1 mM phenylmethanesulfonyl fluoride (PMSF), 1 *μ*g/mL aprotinin, 1 *μ*g/mL leupeptin, 1 *μ*g/mL pepstatin A, 2 mM sodium fluoride, and 1 mM sodium orthovanadate. The cell lysate was centrifuged at 9,500 ×g for 10 min following which a protein extract was obtained from the supernatant. Protein concentration was measured using the PRO-MEASURE assay kit. Extracts containing equal amounts of protein (40 *μ*g) were mixed with 1x SDS-PAGE loading buffer (Biosesang Inc., Korea) and boiled for 5 min, separated by using 10% SDS-PAGE, and further transferred onto polyvinylidene fluoride (PVDF) membranes. The membranes were blocked with blocking buffer (10 mM Tris-HCl (pH 7.5), 150 mM NaCl, 0.1% Tween 20, and 5% nonfat dry milk) for 2 h at room temperature and then incubated with primary antibodies for 18 h at 4°C. After washing with TBST (10 mM Tris-HCl (pH 7.5), 150 mM NaCl, 0.1% Tween 20), the membranes were incubated with the secondary antibody for 2 h at 4°C. Immunoreactive protein bands were visualized using enhanced chemiluminescence (ECL). *β*-actin was used as a loading control.

### 2.8. Nuclear and Cytoplasm Protein Extraction

Nuclear and cytosolic protein extracts were prepared as previously described [15]. Cells were treated with different concentrations of bee venom prior to LPS stimulation. After incubation, the cells were washed three times with ice-cold PBS, resuspended in lysis buffer A (10 mM HEPES, 10 mM KCl, 2 mM MgCl_2_, 0.1 mM EDTA, 1 mM DTT, 0.1 mM PMSF, 2 *μ*g/mL leupeptin, 2 *μ*g/mL aprotinin, and 2 *μ*g/mL pepstatin), centrifuged, and transferred to cytosolic proteins. After isolating the cytosolic proteins, the pellet was resuspended in lysis buffer B (10 mM HEPES, 50 mM KCl, 300 mM NaCl, 0.1 mM EDTA, 0.1 mM PMSF, 2 *μ*g/mL leupeptin, 2 *μ*g/mL aprotinin, and 10% glycerol) and centrifuged again. The supernatant was transferred to new tubes and was used as the nuclear protein extract.

### 2.9. Immunoprecipitation

BV2 microglial cells were pretreated with bee venom and then stimulated with LPS for 30 min. After 15 and 30 min, stimulated BV2 microglial cells were resuspended in IP buffer (50 mM Tris-HCl (pH 7.5), 20 mM NaF, 25 mM beta-glycerophosphate (pH 7.5), 120 mM NaCl, 2% NP-40, and various protease inhibitors). This was followed by addition of primary antibody and incubation with gentle rocking overnight at 4°C. After the overnight incubation, protein A agarose beads were added, and the pelleted beads were washed five times with 500 *μ*L of 1x IP buffer. The pellet was resuspended in Laemmli sample buffer (Bio-Rad) and loaded on an SDS-PAGE gel. After electrophoresis, the proteins were transferred onto a PVDF membrane. The membrane was blocked with 5% defatted milk and then incubated with the primary and secondary antibodies. Immunoreactive bands were visualized using enhanced ECL.

### 2.10. Luciferase Assay

BV2 microglial cells were cultured in 24-well plates for 24 h and then transfected in triplicate with TK-renilla (pRL-TK) and NF-*κ*B firefly luciferase (pNF-*κ*B-Luc) constructs (Stratagene, La Jolla, CA, USA) by using Lipofectamine*™* 2000 according to the manufacturer's instructions. Briefly, transfected cells were pretreated with bee venom for 30 min and then stimulated with LPS for 6 h. Next, the cells were washed twice with ice-cold PBS and then 150 *μ*L of 1x passive lysis buffer was added. After centrifugation at 12,000 ×g for 5 min at 4°C, a 10 *μ*L aliquot of the supernatant was analyzed using a Glomax luminometer (Promega, Madison, WI, USA). NF-*κ*B luciferase activity was measured using a luciferase assay system according to the manufacturer's instructions. Luciferase activity was normalized to renilla luciferase activity.

### 2.11. Statistical Analysis

One-way ANOVA with a post hoc Dunnett's multiple comparison and Student's *t-*test was used to determine the statistical significance of differences between the experimental and control groups. *p* values of 0.05 or less were considered statistically significant. Data represent the means ± SEM of three experiments conducted in triplicate.

## 3. Results

### 3.1. Inhibitory Effect of Bee Venom on Nitric Oxide Production in LPS-Stimulated BV2 Microglial Cells

**Figure 2:**
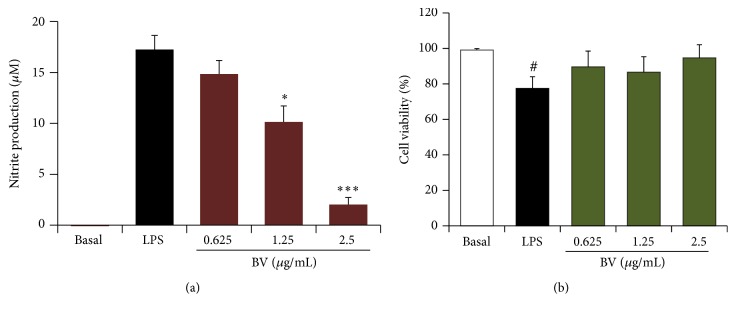
Bee venom inhibits lipopolysaccharide- (LPS-) induced nitric oxide (NO) production in BV2 microglia. (a) The effect of bee venom on the LPS-induced NO production in BV2 cells. (b) The effect of bee venom on the cell viability of LPS-stimulated BV2 cells. BV2 microglial cells were pretreated with different concentrations of bee venom extract (0.625 *μ*g/mL–2.5 *μ*g/mL) for 30 min and then incubated with LPS (0.1 *μ*g/mL) for 24 h. NO production was determined in the cell supernatants as described in Section 2. After determination of NO production, the viability was immediately examined using the 3-(4,5-dimethylthiazol-2-yl)-2,5-diphenyltetrazolium bromide (MTT) assay as described in Section 2. The data are presented as mean ± standard error (SEM), and the experiments were repeated three to five times. ^*∗*^ *p* < 0.05  and  ^*∗∗∗*^ *p* < 0.001 versus LPS alone. ^#^ *p* < 0.05 versus basal.

Nitric oxide (NO) not only acts as an inflammatory mediator and a regulator of inflammatory action, but also has detrimental effects on host tissues [16]. Activated BV2 microglial cells induce iNOS expression and NO* production* in neuronal inflammation. Therefore, we initially examined whether bee venom extract affected NO production in LPS-activated BV2 cells. It was observed that LPS treatment prominently increased NO production (17.3 ± 1.4 *μ*M) in BV2 microglial cells compared to untreated cells (Figure 2(a)), and this increase was markedly reduced by bee venom pretreatment in a concentration-dependent manner. Next, we evaluated the cytotoxicity of bee venom and found that it did not display any cytotoxicity even at 2.5 *μ*g/mL in the presence of LPS stimulation (Figure 2(b)). Therefore, it was concluded that the inhibitory effect of bee venom was not due to cytotoxicity.

### 3.2. Inhibitory Effect of Bee Venom on the mRNA and Protein Expression of iNOS and COX-2 in BV2 Microglial Cells

**Figure 3:**
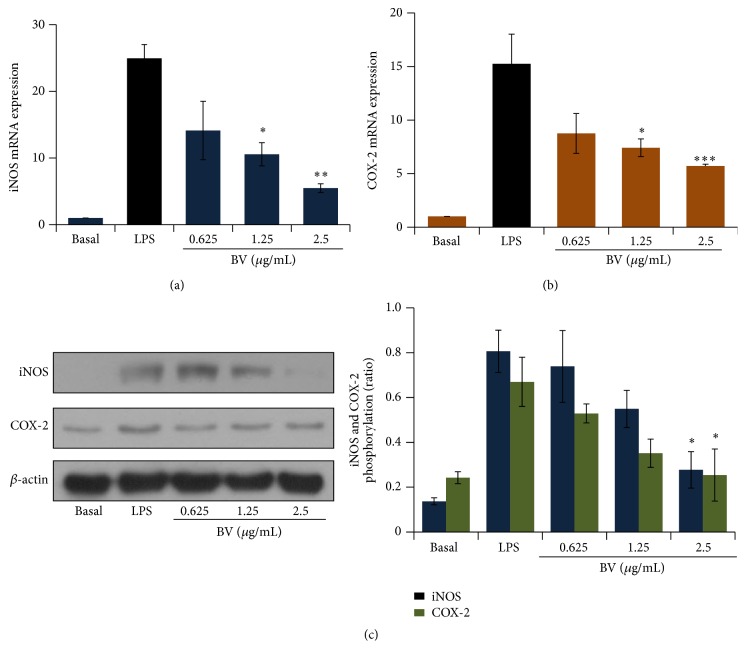
Bee venom inhibits the expression of inducible NO synthase (iNOS) and cyclooxygenase-2 (COX-2) mRNA and protein in LPS-activated BV2 microglia. (a) The effect of bee venom on* iNOS* mRNA expression in LPS-stimulated BV2 cells. (b) The effect of bee venom on* COX-2* mRNA expression in LPS-stimulated BV2 cells. (c) The effect of bee venom on iNOS and COX-2 protein expression in LPS-stimulated BV2 cells. BV2 microglial cells were pretreated with bee venom (0.625–2.5 *μ*g/mL) or vehicle for 30 min and then stimulated with LPS (0.1 *μ*g/mL) for 24 h. Then, total RNA was prepared (to assess mRNA expression), or protein was extracted as described in Section 2. ((a) and (b)) The levels of* iNOS* and* COX-2* mRNA expression were determined using quantitative real-time polymerase chain reaction (PCR). The protein concentration of the cell extracts was determined with PRO-MEASURE (iNtRON Biotechnology, Korea). The protein separation and immunoblot procedures are described in Section 2. The data are presented as mean ± SEM, and experiments were performed three to five times. Representative images of experiments performed at least in triplicate are shown. ^*∗*^ *p* < 0.05, ^*∗∗*^ *p* < 0.01, and ^*∗∗∗*^ *p* < 0.001 versus LPS alone.

NO, which has a crucial role in the initiation of inflammation, is produced in high amounts by iNOS [17]. To determine whether the inhibitory effect of bee venom on NO production was due to decreased iNOS expression, we measured iNOS mRNA and protein expression by real-time PCR and immunoblotting, respectively. iNOS was highly expressed following LPS stimulation (Figures 3(a) and 3(c)). However, this enhanced mRNA and protein expression was greatly suppressed by bee venom pretreatment in a concentration-dependent manner. We then investigated whether bee venom also had an effect on COX-2 mRNA and protein expression in BV2 microglial cells. It was observed that bee venom treatment inhibited the expression of COX-2 mRNA and protein in a dose-dependent manner (Figures 3(b) and 3(c)).

### 3.3. Inhibitory Effect of Bee Venom on LPS-Induced mRNA Expression of Proinflammatory Cytokines in BV2 Microglial Cells

**Figure 4:**
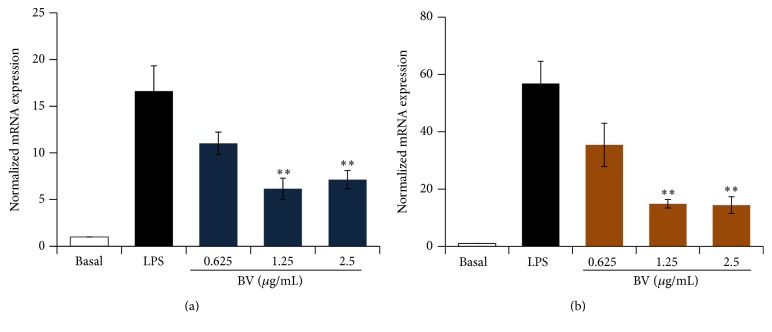
Bee venom inhibits the expression level of tumor necrosis factor-*α* (*TNF-α*) and interleukin-6 (*IL-6*) mRNA in LPS-stimulated BV2 microglia. Dose-dependent inhibition of* TNF-α* (a) and* IL-6* (b) mRNA expression were assessed using quantitative real-time PCR. BV2 cells were pretreated with bee venom for 30 min, and then 0.1 *μ*g/mL LPS was added and the cells were incubated for an additional 24 h. The total RNA preparation and real-time PCR were performed as described in Section 2. GAPDH was used as an internal control, and relative expression levels of* TNF-α* and* IL-6* mRNA were calculated by normalization to* GAPDH*. The data are presented as mean ± SEM of three to five experiments. ^*∗∗*^ *p* < 0.01 versus LPS alone.

Microglia cell activation upregulates proinflammatory cytokines such as TNF-*α* and IL-6, and these can be toxic to neurons and other glial cells. In addition, activated microglial cells contribute to the development of neurodegenerative diseases in the CNS. Therefore, these cytokines merit interest as potential targets in the treatment of neurodegenerative disorders [18]. Following LPS stimulation, TNF-*α* and IL-6 were highly expressed (Figures 4(a) and 4(b)). When BV2 microglial cells were pretreated with bee venom (0.625, 1.25, and 2.5 *μ*g/mL) and then stimulated with LPS (0.1 *μ*g/mL) for 24 h, a significant inhibition of proinflammatory cytokine expression was detected. These findings suggested that bee venom could disrupt the expression of IL-6 and TNF-*α* at the transcriptional level.

### 3.4. Inhibitory Effect of Bee Venom on LPS-Induced NF-*κ*B Activation, I*κ*B-*α* Degradation, and IKK*α*/*β* Phosphorylation

**Figure 5:**
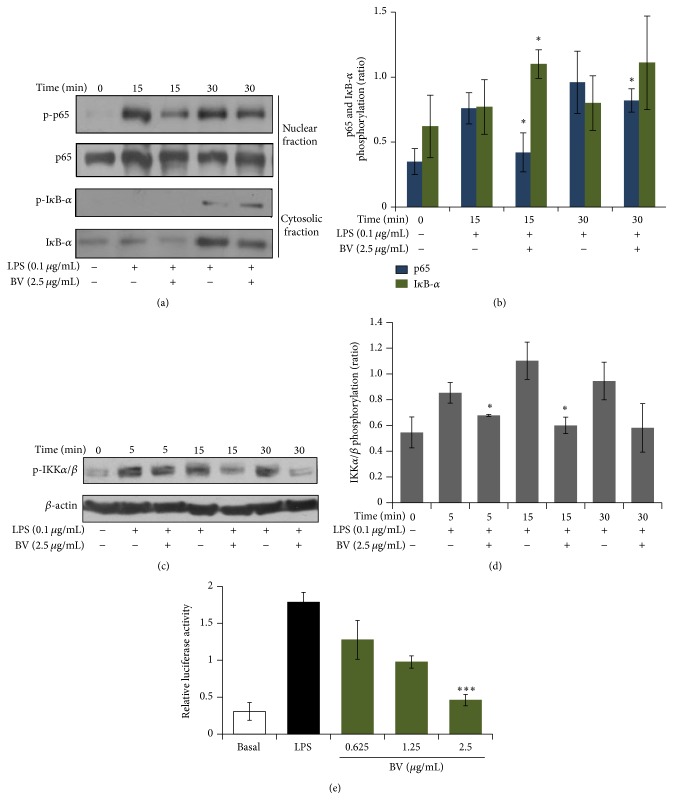
Inhibition of degradation of I*κ*B-*α*, phosphorylation of IKK*α*/*β*, nuclear translocation of the p65 subunit of NF-*κ*B/Rel, and NF-*κ*B transcriptional activity by bee venom in LPS-stimulated BV2 microglia. The inhibitory effect of bee venom on the nuclear translocation of the p65 subunit of NF-*κ*B/Rel, degradation of I*κ*B-*α* (a), phosphorylation of IKK*α*/*β* (b), and NF-*κ*B transcriptional activity (c). BV2 cells were pretreated with bee venom or vehicle for 30 min and then stimulated with 0.1 *μ*g/mL LPS for the indicated times. The protein extraction and SDS-PAGE methods are described in Section 2. The *β*-actin was used as an internal loading control for the immunoblot analysis. The effect of bee venom on NF-*κ*B transcriptional activity was determined by firefly luciferase activity by using a luminometer. ^*∗*^ *p* < 0.05 and ^*∗∗∗*^ *p* < 0.001 versus LPS alone.

The transcription factor NF-*κ*B is activated by the degradation of phosphorylated I*κ*B-*α*, which is phosphorylated by I*κ*B-*α* kinase (IKK) [19]. To examine the effect of bee venom on the degradation of I*κ*B-*α* and the phosphorylation of IKK*α*/*β*, BV2 microglial cells were stimulated with LPS in the presence or absence of bee venom. LPS treatment alone augmented the phosphorylation of NF-*κ*B at 15 and 30 min (Figure 5). However, bee venom pretreatment decreased NF-*κ*B translocation from the cytoplasm to the nucleus, I*κ*B-*α* degradation, and IKK*α*/*β* phosphorylation in a time-dependent manner. Next, we determined whether bee venom could reduce NF-*κ*B transcriptional activity. It was observed that bee venom extract significantly repressed NF-*κ*B activity in a concentration-dependent manner, suggesting that NF-*κ*B is a critical target in bee venom-mediated anti-inflammatory action (Figure 5(e)).

### 3.5. Inhibitory Effect of Bee Venom on LPS-Induced Transforming Growth Factor-*β*- (TGF-*β*-) Activated Kinase 1 and MAPK Phosphorylation

**Figure 6:**
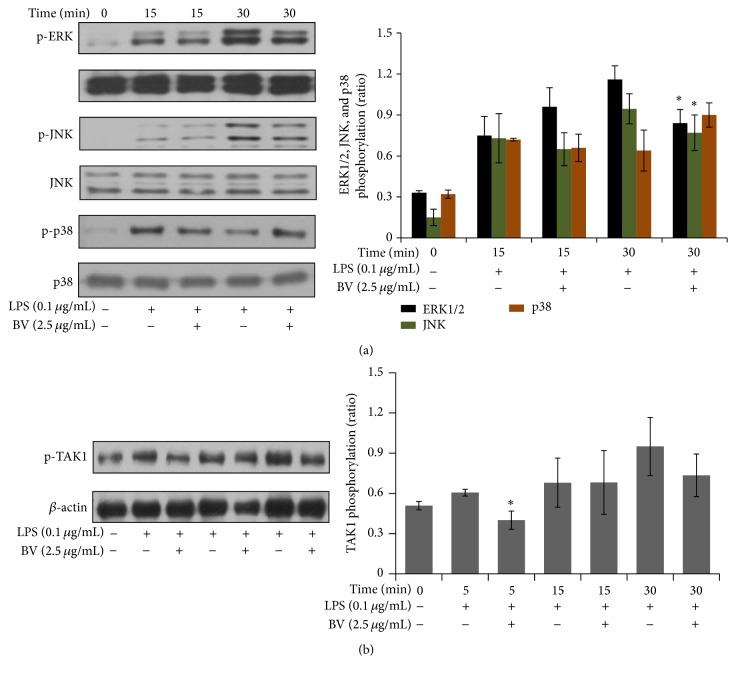
Bee venom modulates MAPK and TAK1 phosphorylation in LPS-stimulated BV2 microglia. The inhibitory effects of bee venom on the phosphorylation of extracellular-signal regulated kinase 1/2 (ERK1/2), c-jun N-terminal kinase (JNK), p38 mitogen-activated protein kinase (p38-MAPK) (a), and TGF-*β*-activated kinase 1 (TAK1) (b) in LPS-activated microglial cells. BV2 cells were pretreated with bee venom or vehicle for 30 min and then incubated with LPS for the indicated times. The protein extraction and protein separation by SDS-PAGE are described in Section 2. *β*-actin was used as an internal loading control for the immunoblot analysis. Representative images of experiments performed at least in triplicate are shown. ^*∗*^ *p* < 0.05 versus LPS alone.

TGF-beta activated kinase 1 (TAK1) functions as an upstream signaling molecule of NF-*κ*B. An activated TAK1 complex will phosphorylate critical kinases, including p38 MAPK, c-jun N-terminal kinase (JNK), and IKK, which activates NF-*κ*B [20]. Since we learnt that bee venom suppressed NF-*κ*B activation, we hypothesized that bee venom might inhibit the augmentation of NF-*κ*B translocation via TAK1 phosphorylation in the LPS-TLR4 signaling pathway. To determine whether bee venom disrupts MAPKs and TAK1 phosphorylation, we evaluated the levels using immunoblot analysis. Bee venom attenuated LPS-induced ERK1/2 and JNK phosphorylation but not p38 MAPK phosphorylation with statistical significance at 30 min (Figures 6(a) and 6(b)). In addition, bee venom significantly inhibited LPS-induced TAK1 phosphorylation at earlier activation of 5 min (Figure 6(b)).

### 3.6. Inhibitory Effect of Bee Venom on the Interaction between MyD88 and Its Downstream Signaling Molecules

**Figure 7:**
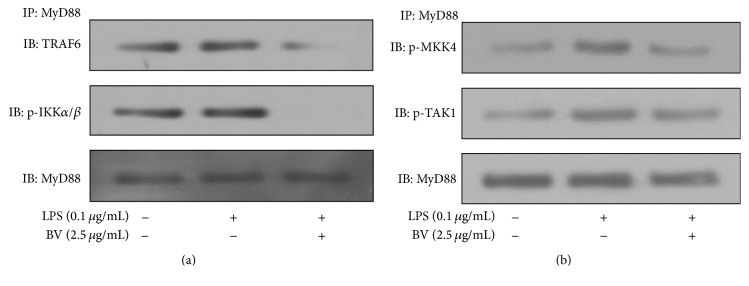
The interaction of MyD88 with either TRAF6 and IKK*α*/*β* or MKK4 and TAK1 was diminished by bee venom treatment in LPS-stimulated BV2 microglia. The inhibitory effects of bee venom on the interaction of MyD88 with TRAF6 (a) and IKK*α*/*β* or MKK4 and TAK1 (b) in LPS-activated microglial cells are shown. BV2 cells were pretreated with bee venom or vehicle for 30 min and then incubated with LPS for the indicated times. The protein extraction, immunoprecipitation with MyD88 antibody, and protein separation by SDS-PAGE are described in Section 2. Representative images of experiments performed at least in triplicate are shown.

MyD88 is an important component of signal transduction in TLR4 activation. Upon binding of LPS to TLR4, an adapter molecule MyD88 was recruited into the receptor and then subsequently recruits IL-1 receptor-associated kinase (IRAK1) into the TLR-MyD88 complex. Interaction between MyD88 and IRAK1 leads to TRAF6 phosphorylation, which activates the downstream signaling pathway [21]. It has recently been reported that the MyD88-dependent pathway is involved in activation of LPS and expression of inflammatory cytokines in BV2 microglial cells [22, 23]. Therefore, we hypothesized that the anti-inflammatory effect of bee venom might be via inhibition of the MyD88-dependent pathway. In the present study, we examined the interaction between MyD88 and other signaling molecules, TRAF6, IKK*α*/*β*, MKK4, and TAK1,* using protein complex immunoprecipitation technique*. The association of the tested signaling molecules, including TRAF6, IKK*α*/*β*, MKK4, and TAK1, with MyD88 was greatly diminished after 30 min of bee venom treatment. This suggests that the bee venom diminished the signaling pathway at earlier components in the Myd88-dependent pathway of LPS-TLR4 activation (Figure 7).

## 4. Discussion

Several reports have shown that bee venom exhibits antineuroinflammatory activity [9, 24, 25]. However, the signaling pathways that govern this activity are still not clear. Therefore, we explored the anti-inflammatory activities of bee venom and the actual signaling pathways that inhibit LPS-induced inflammation in BV2 microglial cells. Our data indicated that bee venom could neutralize LPS-induced inflammatory responses in microglial cells through a MyD88-dependent pathway. To be precise, bee venom extract significantly inhibited LPS-induced NO production and expression of the proinflammatory cytokines TNF-*α* and IL-6 in BV2 cells in a concentration-dependent manner. In addition, bee venom was found to inhibit the transcriptional activity of NF-*κ*B. Analysis of the downstream signaling molecules of LPS-TLR4 showed that the MyD88-IRAK1-TRAF6-TAK1-MKK4 pathway and the MyD88-TAK1-IKK*α*/*β* pathway were modulated by bee venom treatment. The major finding of this study was that the MyD88-IRAK1-TRAF6-TAK1-MKK4 and MyD88-TAK1-IKK*α*/*β* pathways are the novel inhibitory mechanisms underlying bee venom-mediated inhibition of the LPS-induced inflammatory response in BV2 microglial cells.

Microglial cells are resident immune cell population in the CNS that are intensely responsive to brain injury and neuronal disorders, and when these occur, they become rapidly activated. This reaction is a part of the normal response to maintain brain homeostasis [5]. However, overactivated microglia can cause neuronal death and neurodegenerative diseases, including Parkinson's disease, Alzheimer's disease, and trauma. Therefore, regulation of microglial activation by bee venom might be a useful option for the prevention or therapy against neurodegenerative diseases [26].

LPS, a macromolecular complex found in the cell walls of gram-negative bacteria, is an endotoxin that is covalently linked to a lipid A [5]. Several reports have shown that LPS can strongly activate microglia. Activated microglia release a large variety of inflammatory mediators, including proinflammatory cytokines, chemokines, and neurotoxic factors [27] In particular, NO is an interesting target molecule due to its Janus-like ability to mediate the inflammatory response by inhibiting or prompting the inflammatory response through several different pathways. Exposure to LPS or other stimuli induces the expression of iNOS in immune cells, and iNOS can constantly produce a large amount of NO [28, 29]. In the present study, we found that LPS-induced NO production was significantly attenuated by bee venom treatment, and this effect could emanate from its inhibitory effect on iNOS.

TLRs are members of the IL-1R/TLR superfamily that play crucial roles in inflammatory responses to invading pathogens by recognizing specialized microbial components. Among several TLR subtypes, TLR4 is required for LPS stimulation and is involved in host defense against inflammation, apoptosis, and cancer [30]. MyD88 is an adaptor protein that possesses a TIR domain in its C-terminus and a death domain in its N-terminus. The association of its TIR domain with TLRs is responsible for transmitting the intracellular signal from TLR4 after LPS stimulation [31]. Upon TLR4 activation, MyD88 recruits IRAK4 and then induces IRAK1 phosphorylation. Phosphorylated IRAK associates with TRAF6, which leads to activation of JNK and NF-*κ*B [32]. Interaction of TAK1 with ubiquitinated TRAF6 leads to TAK1 activation. TAK1 is an MAP3 K that is activated by TGF-*β* and plays a critical role in relaying the signal that promotes inflammation-related cytokine expression [33]. Our data clearly showed that bee venom blocked LPS-activated inflammatory responses in a MyD88-dependent manner (Figure 7).

The transcription factor NF-*κ*B has a critical role in the innate inflammatory response and is a key mediator that is responsible for several key biological processes such as immune and inflammatory responses. NF-*κ*B is activated in the macrophages upon stimulation with proinflammatory cytokines [34]. Activated IKK*α*/*β* causes degradation of I*κ*B-*α* and translocation of NF-*κ*B to the nucleus [35]. In addition, bee venom abolished the translocation of NF-*κ*B into the nucleus, thereby deactivating the NF-*κ*B transcriptional activity. This may be due to blockade of the interaction between MyD88 and IKK*α*/*β* (Figure 6(b)).

**Figure 8:**
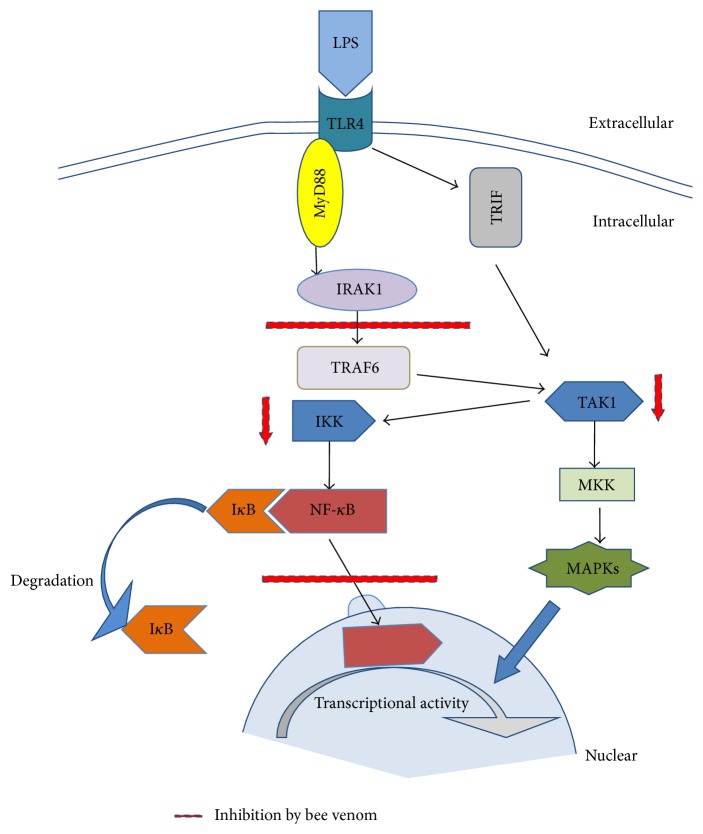
Graphical summary of anti-inflammatory activity of bee venom in LPS-activated BV2 glial cells.

In summary, we found that bee venom diminished LPS-induced proinflammatory cytokines, iNOS, and COX-2 expression. In addition, the phosphorylation of the three MAPKs was significantly attenuated following bee venom treatment. Interestingly, the association of MyD88 with IRAK and TRAF6 was disrupted (at 15 min) by bee venom pretreatment. Moreover, the interaction of MyD88 with MKK3/4 and IKK*α*/*β* was also significantly inhibited at 30 min. Therefore, bee venom limits LPS-induced neuroinflammation through inhibition of the association of MyD88 with TRAF6 and IRAK1, which inhibits activation of downstream signaling molecules (Figure 8). Bee venom has been shown to prevent LPS-induced I*κ*B-*α*/*β* phosphorylation, which, in turn, inhibits the translocation of NF-*κ*B and the MAPK-dependent pathways. These results support our rationale of the novel mechanism underlying bee venom's anti-inflammatory effects in BV2 microglia.
